# The safety of antithrombotic therapy in patients with cerebral microbleeds and cardiogenic cerebral embolism due to nonvalvular atrial fibrillation

**DOI:** 10.1186/s12872-019-1046-y

**Published:** 2019-04-02

**Authors:** Jiayu Wang, Jia Zhang, Yuan Shen, Xiaowei Xu

**Affiliations:** 1 Department of Cardiovascular, Beijing Tiantan Hospital, Capital Medical University, No. 119 South 4th Ring West Road, Beijing, 100070 Fengtai District China; 2 Department of Neurology, Beijing Tiantan Hospital, Capital Medical University, Beijing, China

**Keywords:** Cardio embolism, Cerebral microbleeds, Nonvalvular atrial fibrillation, Antithrombotic therapy

## Abstract

**Background:**

The impact of cerebral microbleeds on the safety of antithrombotic therapy has recently received considerable attention. We investigated the safety of antithrombotic therapy in patients with cerebral microbleeds and cardiogenic cerebral embolism caused by nonvalvular atrial fibrillation.

**Methods:**

This retrospective study enrolled patients with acute cardiogenic cerebral embolism due to nonvalvular atrial fibrillation in the stroke unit of the Department of Neurology at the Beijing Tiantan Hospital, the Capital Medical University, from January 2015 to January 2018. General clinical data, magnetic resonance imaging data, and data regarding the use of antithrombotic medications were collected. The main adverse events were cerebral hemorrhage and all-cause death.

**Results:**

According to the susceptibility-weighted imaging sequence, patients were divided into a cerebral microbleeds group and a non-cerebral microbleeds group. Patients with cerebral microbleeds were more likely to be male and to have a history of hypertension and diabetes, and they were less likely to have received anticoagulant therapy (49.1% vs. 71.3%, *P* = 0.001). However, no significant differences were found in the event-free time, the occurrence of cerebral hemorrhage events and all-cause death. Cox regression analysis showed that the risk of all-cause death in patients with a cerebral hemorrhage history and cerebral microbleeds increased 2.773-fold (HR = 2.773, 95%CI 1.056–7.280, *P* = 0.019), and the risk of a cerebral hemorrhage event in patients with cerebral microbleeds and a hypertension history (HR = 3.451, 95%CI 1.947–6.119, *P* = 0.045) or a cerebral hemorrhage history (HR = 2.443, 1.078–5.536, *P* = 0.006) was increased 3.451-fold and 2.443-fold, respectively.

**Conclusions:**

Antithrombotic therapy in patients with CMBs and cardiogenic cerebral embolism due to nonvalvular atrial fibrillation did not have increased risks of a cerebral hemorrhage event and all-cause death. CMBs were probably not a crucial predictor of whether patients were prescribed antithrombotic medicine. Patients with CMBs and a hypertension history or cerebral hemorrhage history should receive a close follow-up after antithrombotic therapy.

## Background

Atrial fibrillation (AF) is a common arrhythmia, and it that significantly increases the incidence of cardiogenic cerebral embolism. In the absence of effective antithrombotic therapy, the risk of cardiogenic cerebral embolism increases by 5-times in patients with AF [1]. Antithrombotic therapy plays a crucial role in primary and secondary prevention of cardiogenic cerebral embolism in patients with AF. Compared with other types of ischemic stroke, cardiogenic cerebral embolism caused by AF is more likely to lead to death or lifelong disability and dependence [1].

Cerebral microbleeds (CMBs) represent one of the imaging manifestations of cerebral small-vessel disease, which is a subclinical lesion of the brain parenchyma characterized by microbleeds [2]. The incidence of CMBs in the general population is approximately 15%, and CMBs are more common in the elderly. The detection rate of CMBs in people over 80 years old is as high as 35.7% [3]. The incidence of CMBs in ischemic stroke patients is approximately 20% [4]. Studies have shown that CMBs not only have an important impact on hemorrhagic transformation but are also an independent risk factor for new ischemic stroke or transient ischemic attack and stroke recurrence [2–4]. Therefore, the role of CMBs is important for the prevention and treatment of various strokes [5].

Previous studies [6] have shown that the incidence of CMBs in patients with AF is significantly higher than that in patients without AF, and the presence of CMBs is associated with an increased risk of stroke and death in patients with AF [7, 8]. Thrombolysis, antiplatelet therapy or anticoagulant therapy is generally applied for primary and secondary prevention of ischemic stroke. For patients with ischemic stroke and CMBs, whether antithrombotic therapy increases the risk of cerebral hemorrhage has not been fully confirmed. CMBs may increase the risk of antithrombotic therapy-associated cerebral hemorrhage, and nonvalvular atrial fibrillation (NVAF) patients have a clear need for antithrombotic therapy and may even require high-intensity antithrombotic therapy. Therefore, the potential risks and possible benefits of antithrombotic therapy for these patients should be carefully considered.

One study has [9] shown that in NVAF patients with acute ischemic stroke, the long-term mortality rate of patients with CMBs is higher than that of patients without CMBs, and the presence and number of CMBs predict cardiovascular and stroke-related mortality. However, in clinical guidelines [10], antithrombotic therapy is not a contraindication for AF patients with CMBs. Therefore, patients with both NVAF and CMBs should be evaluated for the benefit of stroke prevention therapy and the cerebral hemorrhage risk. Although ischemic stroke risk stratification methods in AF patients have been established, the indictors for risk stratification and cerebral hemorrhage assessment to guide clinical practice are not clear.

The impact of CMBs on the safety of antithrombotic therapy has recently received considerable attention. The objective of this study was to investigate the safety of antithrombotic therapy in patients with CMBs and cardiogenic cerebral embolism caused by NVAF.

## Methods

### Patient selection

This retrospective study enrolled patients with acute cardiogenic cerebral embolism due to NVAF in the stroke unit of the Department of Neurology at the Beijing Tiantan Hospital, the Capital Medical University, from January 2015 to January 2018. Cardio embolism was considered in the etiology of acute cerebral infarction in all patients according to the traditional classification of subtypes of ischemic stroke (the Trial of Org 10,172 in Acute Stroke Treatment, TOAST).

The exclusion criteria were: (a) incomplete data; (b) death during hospitalization; (c) acute cardiogenic cerebral embolism with hemorrhagic transformation that could not be treated with antithrombotic therapy; (d) refusal to use antithrombotic therapy; (e) the presence of cognitive deficits; (f) the presence of serious diseases affecting prognosis, such as malignant tumors; (g) a pathogenesis of cardiac embolisms such as a patent foramen ovale, atrial septal defect, atrial myxoma, and infective endocarditis; and (h) the presence of valvular atrial fibrillation.

The study protocol conforms to the ethical guidelines of the Declaration of Helsinki and was approved by the Ethics Committee of the Beijing Tiantan Hospital. The methods were strictly carried out in accordance with the approved guidelines. Informed consent was obtained from all participants or guardians if they were disturbance of consciousness.

### Diagnostic criteria

According to the traditional classification of subtypes of ischemic stroke (TOAST) [11, 12], patients with the following characteristics were considered to have cardio embolism: (a) embolization of different arterial distribution areas, including multiple regions at multiple times; (b) cerebral infarction mainly located in the cortex or cortical tract; and (c) no obvious cerebral artery stenosis.

According to magnetic resonance imaging (MRI, MAGNETOM Skyra 3.0 T, SIEMENS, Germany), the diagnostic criteria [13] for CMBs included low-signal loss foci with a round or oval shape, a clear border, a diameter of 2–10 mm, and a lesion at least halfway surrounded by brain parenchyma on cerebral MRI with the susceptibility-weighted imaging (SWI) sequence. Exceptions included traumatic brain injury; massive axonal injury with similar imaging findings, such as signal loss due to blood flow effects in the vascular space, brain groove area or small blood vessels; hemosiderin deposition in the pia mater; subcortical calcification without hemorrhage; calcification or iron deposition in the basal ganglia; and cerebral vascular malformation, such as telangiectasia and cavernous hemangioma.

According to their anatomy, CMBs were classified based on whether they were located in (a) a brain lobe of the cortex or a subcortical region; (b) a deep brain tissue region, including the basal ganglia, thalamus, internal sac, corpus callosum, deep and paraventricular white matter, brainstem or cerebellum; or (c) both a brain lobe and a deep brain tissue region. According to the number of CMBs identified with the SWI sequence, CMBs were divided into groups of ≤5, 6 to 9 and ≥ 10. Two independent observers who knew the purpose of the study but were blinded to the study design and the results of all investigations evaluated the location and number of CMBs identified with the SWI sequence of MRI.

Cerebral infarction and cerebral hemorrhage in our study mainly referred to two intervals. A cerebral infarction history or cerebral hemorrhage history was defined as cerebral infarction or cerebral hemorrhage diagnosed by neurologists before the current admission. Cerebral hemorrhage occurring after antithrombotic treatment was defined as a cerebral hemorrhage event.

NVAF [14] referred to AF without artificial mechanical valves or moderate to severe mitral stenosis usually caused by rheumatic fever. Paroxysmal AF [14] was defined as AF lasting fewer than seven days, and persistent AF was defined as AF lasting more than seven days. All patients in our study had an AF history or a recent diagnosis of AF after admission. The presence of an AF history indicated that patients were diagnosed definitively by cardiologists and had been receiving medical treatment or underwent AF catheter ablation before the time of hospitalization for this study. A recent diagnosis of AF indicated that patients without a previous AF history were diagnosed during admission according to an electrocardiogram, dynamic electrocardiogram or other heart rhythm detection.

### Clinical data collection

All patients with acute cerebral infarction in the stroke unit underwent a careful medical history evaluation, physical examination, blood laboratory tests, imaging and heart rhythm detection, including computed tomography angiography and magnetic resonance imaging of the cerebral artery and vein, computed tomography and magnetic resonance imaging of the head, transthoracic echocardiography, transesophageal echocardiography, aortic arch ultrasound, carotid ultrasound, ultrasound of the limb arteries and veins, transcranial doppler ultrasound (TCD), a TCD foaming experiment, TCD micro bubble detection, resting 12-lead ECG and a 24-h ambulatory electrocardiogram.

Information concerning general clinical data, MRI data and the use of antithrombotic medications (warfarin, dabigatran, rivaroxaban, aspirin, or clopidogrel) was collected at the time of admission. General clinical data included gender, age, hypertension and diabetes history, atrial fibrillation history or a new diagnosis of AF, cerebral infarction (CI) history, cerebral hemorrhage (CH) history and smoking. The National Institutes of Health stroke scale (NIHSS) score [15], atrial fibrillation stroke risk CHA_2_DS_2_VASc score [14], and bleeding risk HAS-BLED score [14] were collected at the same time. NIHSS scores of 1–4 indicated mild strokes, 5–15 indicated moderate strokes, 16–20 indicated moderate to severe strokes, and scores higher than 21 indicated severe strokes [16]. Antithrombotic therapy included antiplatelet therapy and anticoagulant therapy. Patients with CHA_2_DS_2_VASc scores ≥2 in males and ≥ 3 in females should be on anticoagulant therapy (warfarin, dabigatran, or rivaroxaban), and those with CHA_2_DS_2_VASc scores < 2 should be on antiplatelet therapy (aspirin or clopidogrel). HAS-BLED scores ≥3 indicated a high risk of bleeding. All patients without a history of AF underwent a resting 12-lead ECG and a 24-h ambulatory electrocardiogram to screen for AF. All patients underwent transesophageal echocardiography to screen for a patent foramen ovale, atrial septal defects, atrial myxoma and heart valve disease. According to the SWI sequence of MRI, patients were divided into a CMBs group and a non-CMBs group.

### Main adverse events

Patient follow-ups were conducted by telephone and required reporting of adverse events related to CH events and all-cause death. A cerebral hemorrhage event was defined as acute extravasation of blood into the brain parenchyma or subarachnoid space with associated neurologic symptoms. All patients with cerebral hemorrhage underwent computed tomography or MRI of the brain and were diagnosed by doctors at admission or in the emergency department. Imaging data and medical records of patients attending in Beijing Tiantan Hospital could be retrieved from the image system and the case system, respectively. Patients attending other hospitals were requested to upload their imaging data and medical records to the follow-up system.

### Statistical analyses

Statistical analyses were performed using SPSS for Windows, version 18.0. Normally distributed continuous variables were expressed as the mean ± standard deviation and compared using Student’s t-test. Non-normally distributed continuous variables were expressed as the medians (upper quartile, lower quartile) and compared using the rank sum test. Normality was tested using the Shapiro–Wilk test. Categorical variables were expressed as percentages, and the χ2 test or Fisher’s exact test was used for comparisons between the two groups. Cox regression analyses were performed to identify variables with predictive value for all-cause death and CH events. All statistical tests were run as two-sided tests. For all tests, *P* < 0.05 was considered statistically significant.

## Results

### Clinical characteristics by CMBs

A total of 232 patients were enrolled in the study, including 110 patients with CMBs and 122 patients without CMBs. No significant differences were found in age, smoking, CI history, CH history, NHISS scores and HAS-BLED scores. Patients with CMBs were more likely to be male and to have a history of hypertension and diabetes, and they were less likely to have been treated with anticoagulant therapy (49.1% vs. 71.3%, *P* = 0.001). Eighty-eight patients were treated with dabigatran 110/150 mg twice a day and 9 patients were treated with 15/20 mg once a day. Fifty-five patients were treated with aspirin 100 mg once a day and 36 patients were treated with clopidogrel 75 mg once a day according to the CYP2C19 genotype. However, no significant difference was found in the use of anticoagulant drugs or antiplatelet drugs between the groups. The CHA_2_DS_2_VASc score was higher in patients without CMBs (Table 1).

**Table 1:** Comparison of general clinical data between the two groups

	CMBs group *n* = 110	non-CMBs group *n* = 122	t orχ^2^	*P* value
Patient characteristics				
Male gender (%)	81 (73.6)	74 (60.7)	4.396	0.036
Age (years)	68.1 ± 11.4	68.0 ± 13.0	0.039	0.969
Smoking (%)	36 (32.7)	49 (40.2)	1.378	0.240
Hypertension history (%)	81 (73.6)	72 (59.0)	5.506	0.019
Diabetes history (%)	48 (43.6)	37 (30.3)	4.413	0.036
AF status			6.380	0.012
AF history (%)	56 (50.9)	82 (67.2)		
New diagnosis of AF (%)	54 (49.1)	40 (32.8)		
AF type			0.045	0.833
Paroxysmal AF (%)	40 (36.4)	46 (37.7)		
Persistent AF (%)	70 (63.6)	76 (62.3)		
CI history (%)	22 (20.0)	21 (17.2)	0.298	0.585
CH history (%)	4 (3.6)	2 (1.6)	0.916	0.339
NIHSS score	6.3 ± 5.2	7.1 ± 5.6	−0.864	0.389
CHA_2_DS_2_VASc score	5.0 ± 1.2	5.4 ± 1.2	−2.922	0.004
HAS-BLED score	1.6 ± 0.4	1.5 ± 0.5	0.152	0.880
Antithrombotic therapy			11.981	0.001
Anticoagulant therapy	54 (49.1)	87 (71.3)	1.180	0.554
Warfarin (%)	14 (12.7)	30 (24.6)		
Dabigatran (%)	36 (32.7)	52 (42.6)		
Rivaroxaban (%)	4 (3.6)	5 (4.1)		
Antiplatelet therapy	56 (50.9)	35 (28.7)	0.005	0.946
Aspirin (%)	34 (30.9)	21 (17.2)		
Clopidogrel (%)	22 (20.0)	14 (11.5)		

### Main adverse events

The time from event-free status to the main adverse events was (22.4 ± 13.4) months, and no significant difference was found in the event-free time between the groups. Among all patients, 12.7% (*n* = 14) of the patients with CMBs died, and 16.4% (*n* = 18) of the patients with CMBs experienced a CH event. No significant differences were found in all-cause death and CH events between the two groups (Table 2).

**Table 2:** Comparison of the main adverse events between the two groups

	CMBs group *n* = 110	non-CMBs group *n* = 122	t or χ^2^	*P* value
Event-free time (months)	21.4 ± 12.7	23.4 ± 14.0	−1.101	0.272
CH events (%)	18 (16.4)	13 (10.7)	1.628	0.202
All-cause death (%)	14 (12.7)	13 (10.7)	0.241	0.623

### Main adverse events by the region and number of CMBs

Among the patients with CMBs, 45.5% (*n* = 50) had deep brain tissue CMBs, and 36.4% (*n* = 40) had ≥10 CMBs. The comparison of the region and number of CMBs is shown in Table 3. No significant differences were found in all-cause mortality and CH events among the different regions and number of CMBs. However, a significant difference was found in anticoagulant therapy; the patients with CMBs in mixed regions and more than 10 CMBs were more likely to have received anticoagulant therapy (Table 4 and Table 5).

**Table 3:** Comparison of the region and number of CMBs

	Region of CMBs			χ^2^	*P* value
	Brain lobe *n* = 42	Deep brain tissue *n* = 50	Mixed regions *n* = 18		
Number of CMBs				16.036	0.003
≤ 5, *n* = 46	20 (47.6)	26 (52.0)	0,0		
6–9, *n* = 24	8 (19.0)	10 (20.0)	6 (33.3)		
≥ 10, *n* = 40	14 (33.3)	14 (28.0)	12 (66.7)

**Table 4:** Comparison of the main adverse events and antithrombotic therapy according to the region of CMBs

	Region of CMBs			χ^2^	*P* value
	Brain lobe *n* = 42	Deep brain tissue* n* = 50	Mixed region *n* = 18		
Antithrombotic therapy					
Anticoagulant therapy (%)	20 (47.6)	20 (40.0)	14 (77.8)	7.617	0.022
Antiplatelet therapy (%)	22 (52.4)	30 (60.0)	4 (32.2)		
CH events (%)	8 (19.0)	6 (12.0)	4 (22.2)	1.368	0.505
All-cause death (%)	2 (4.8)	8 (16.0)	4 (22.2)	4.342	0.114

**Table 5:** Comparison of the main adverse events and antithrombotic therapy according to the number of CMBs

	Number of CMBs			χ^2^	*P* value
	≤5 *n* = 46	6–9 *n* = 24	≥10 *n* = 40		
Antithrombotic therapy					
Anticoagulant therapy (%)	18 (39.1)	8 (33.3)	28 (70.0)	11.208	0.004
Antiplatelet therapy (%)	28 (60.9)	16 (66.7)	12 (30.0)		
CH events (%)	6 (13.0)	2 (8.3)	10 (25.0)	3.681	0.159
All-cause death (%)	8 (17.4)	2 (8.3)	4 (10.0)	1.586	0.453

### Predictors of all-cause death and cerebral hemorrhage

A multivariate analysis of the following variables was performed: CMBs, male gender, smoking, hypertension history, diabetes history, CI history, CH history, use of anticoagulant therapy and NIHSS score ≥ 5. The analysis using a Cox regression model revealed that smoking, CH history, use of anticoagulant therapy and an NIHSS score ≥ 5 were predictors of all-cause death and that smoking, hypertension history, CH history and an NIHSS score ≥ 5 were risk factors for a CH event (Table 6). Considering the interaction between CMBs and other factors influencing the main adverse events, another multivariate analysis of main adverse events stratified by CMBs showed that a CH history and the presence of CMBs increased the risk of all-cause death, and a hypertension history or CH history and the presence of CMBs respectively increased the risk of CH events (Table 7).

**Table 6:** The Cox regression model of the main adverse events

Variable	All-cause death			CH events		
	Hazard Ratio	95% confidence interval	P value	Hazard Ratio	95% confidence interval	*P* value
CMBs	1.467	0.532–4.044	0.458	2.349	0.945–5.840	0.066
Male gender	0.714	0.263–1.944	0.510	0.854	0.331–2.206	0.745
Smoking	1.613	1.117–2.328	0.036	1.413	0.951–2.102	0.090
Hypertension history	1.769	0.538–5.814	0.347	2.840	1.886–4.276	0.025
Diabetes history	1.252	0.453–3.459	0.665	1.251	0.470–3.327	0.654
CI history	1.819	0.615–5.380	0.279	0.633	0.174–2.301	0.488
CH history	4.178	2.336–7.472	0.026	3.028	1.524–6.017	0.038
Anticoagulant therapy	0.690	0.573–0.832	0.003	1.724	0.664–4.772	0.263
NIHSS score ≥ 5	1.613	1.372–1.896	< 0.001	2.376	1.236–4.569	0.043

**Table 7:** Multivariate Cox regression model of the main adverse events stratified by CMBs

	All-cause death				CH events			
	non- CMBs	CMBs	*P* value	*P* interaction	non- CMBs	CMBs	*P* value	*P* interaction
Gender				0.718				0.447
Male	1	1.099 (0.398–3.034)	0.885		1	2.228 (0.785–6.326)	0.132	
Female	1	1.819 (0.603–5.931)	0.275		1	1.712 (0.575–5.096)	0.334	
Smoking				0.143				0.656
Yes	1	0.679 (0.375–1.233)	0.300		1	1.219 (0.304–4.884)	0.780	
No	1	1.940 (0.830–4.534)	0.126		1	2.075 (0.886–4.858)	0.894	
Hypertension history				0.054				0.034
Yes	1	0.856 (0.748–4.605)	0.182		1	3.451 (1.947–6.119)	0.045	
No	1	0.715 (0.236–2.165)	0.357		1	0.658 (0.135–3.210)	0.605	
Diabetes history				0.224				0.183
Yes	1	2.004 (0.638–6.295)	0.234		1	2.388 (0.657–8.683)	0.186	
No	1	0.563 (0.150–2.109)	0.394		1	1.437 (0.560–3.685)	0.274	
CI history				0.170				0.825
Yes	1	1.471 (0.356–6.079)	0.594		1	2.208 (0.199–24.472)	0.519	
No	1	1.309 (0.532–3.223)	0.558		1	1.602 (0.756–3.394)	0.219	
CH history				0.015				0.018
Yes	1	2.773 (1.056–7.280)	0.019		1	2.443 (1.078–5.536)	0.006	
No	1	1.258 (0.564–2.805)	0.575		1	1.565 (0.751–3.261)	0.232	
Anticoagulant therapy				0.415				0.147
Yes	1	1.476 (0.394–5.526)	0.563		1	2.004 (0.814–4.936)	0.131	
No	1	0.838 (0.329–2.134)	0.711		1	1.492 (0.443–5.024)	0.519	
NIHSS score				0.770				0.448
≤ 4	1	0.852 (0.229–3.178)	0.812		1	2.338 (0.604–9.047)	0.101	
≥ 5	1	2.505 (0.783–8.015)	0.122		1	3.077 (1.071–8.844)	0.037	

## Discussion

AF is an important risk factor for cardiogenic cerebral embolism. The clinical symptoms of cardiogenic cerebral embolism tend to be more severe than those of other ischemic stroke subtypes. Oral anticoagulants have become a critical treatment for primary and secondary prevention of cardiogenic cerebral embolism in patients with AF, reducing their risk by up to approximately 67% [17]. Failure to use anticoagulant therapy was an independent risk factor for overall mortality in AF patients. The main cause of death was associated with recurrent cardiogenic cerebral embolism (61.7%) [18]. Studies [18, 19] have shown that patients with AF and CMBs have increased mortality, and anticoagulant therapy increases the risk of CH event. The impact of CMBs on the safety of antithrombotic therapy was the focus of this study [20].

Relatively few studies have explored the relationship between AF and CMBs. Saito et al. [6] showed that the incidence of CMBs is significantly higher in patients with AF than that in non-AF patients. Kim et al. [21] showed that 33% of patients with cardiogenic cerebral embolism have accompanying CMBs. In our study, the rate of nearly 50% of patients with CMBs was higher than that in a previous study. One possible explanation is that MRI with the SWI sequence was used in each patient to increase the detection rate of CMBs.

Compared to patients without CMBs, patients with ischemic stroke and CMBs have a higher prevalence of hypertension [22]. Studies [22, 23] have suggested that hypertension is a risk factor for CMBs, especially CMBs in deep brain tissue. In our study, patients with CMBs were more likely to have a history of hypertension, which is consistent with previous results. In the multivariate analysis, patients had a hypertension history with CMBs increased the risk of a CH event. The presence of CMBs and a hypertension history was associated with the occurrence of a CH event. Long-term hypertension was one of the important causes of CMBs. The pathological and physiological mechanism may be hyaline degeneration of blood vessels caused by hypertension, which may lead to extravasation of blood and the formation of local microvascular aneurysms. Therefore, antithrombotic therapy should be used more cautiously in this population, and blood pressure should be carefully controlled during antithrombotic therapy.

Without effective anticoagulant therapy, the risk of stroke onset and recurrence is significantly increased in patients with AF, and the CHADS_2_ and CHA_2_DS_2_VASc scores may reflect the risk of ischemic stroke [24]. In the non-CMB group, the CHA_2_DS_2_VASc score was higher than that in the CMB group in our study. One possible reason is that more female patients were included in the non-CMB group. AF was more associated with CMBs in the brain lobes [9]. In our study, the proportions of CMBs in the brain lobes and deep brain tissue regions were similar, and no significant difference was found in all-cause death in patients with different quantities of CMBs. CMBs in the brain lobes were prone to cerebral hemorrhages [25], which are different from the pathophysiological mechanism of vascular hyaline degeneration in deep brain tissue CMBs. This was thought to be associated with β-amyloid-deposition cerebral amyloid angiopathy, which is prone to cerebral hemorrhage. Therefore, patients with more CMBs in the brain lobes should be carefully monitored when receiving double antithrombotic therapy. To rule out the influence of mixed factors, the logistic regression analysis showed that CMBs are not an independent risk factor for cerebral hemorrhage in our study.

Thrombolytic, antiplatelet or anticoagulant therapy is required for both primary and secondary prevention of ischemic stroke. Therefore, for patients with ischemic stroke and CMBs, several items must be addressed, including whether antithrombotic therapy increases the risk of bleeding, whether CMBs can be used as an indicator to predict cerebral hemorrhage after antithrombotic therapy, when AF patients with CMBs should begin antithrombotic therapy, and the appropriate strength of thrombolytic therapy in the acute phase of ischemic stroke.

Study by Biffi et al. [26] showed that aspirin may increase the risk of recurrence in patients with cerebral hemorrhage, especially in the presence of multiple CMBs in the brain lobes. In addition, patients with ischemic stroke with > 5 CMBs should not be treated with antithrombotic therapy because the risk will outweigh the benefits [27]. However, one study [28] suggested that antithrombotic therapy can benefit patients with ischemic stroke and CMBs even if a risk of cerebral hemorrhage exists. In our study, no significant association with cerebral hemorrhage was found in patients with ≥5 CMBs and < 5 CMBs. Furthermore, in the multivariate analysis, CMBs were not a predictor for the main adverse events. However, patients having a hypertension history or CH history with CMBs had a higher risk of a CH event, suggesting that identifying patients with a high risk of bleeding is critical for anticoagulation therapies.

Patients with AF and CMBs should be evaluated for the benefit of stroke prevention and the bleeding risk. Although risk stratification methods in AF patients remain controversial, risk stratification and bleeding assessment may guide clinical practice. Long-term, effective and safe anticoagulant therapy and a stable international normalization ratio in patients with AF are associated with a decreased risk of adverse events. Therefore, SWI-sequence screening for CMBs was recommended for patients with ischemic stroke and AF who receive long-term anticoagulant therapy [29].

At present, non-vitamin K antagonist oral anticoagulants (NOACs) are widely used for the prevention of cardio embolism. Their effectiveness is comparable to that of warfarin, but the associated risk of a CH event is significantly lower than that of warfarin. Two small sample studies in Japan [4, 30] showed that NOACs do not increase the risk of new CMBs. Patients with AF and a high risk of embolism need long-term anticoagulant therapy. A multicenter Italian study of anticoagulant therapy after warfarin-related CH events [31] showed that NOAC treatment after a CH event can reduce systemic thrombotic events and all-cause mortality but does not increase the risk of CH recurrence. In contrast, the clinical outcomes of patients who received antiplatelet therapy or discontinued antithrombotic therapy did not improve significantly. In this study, anticoagulant therapy did not increase the incidence of adverse events in patients with multiple CMBs, and in the multivariate regression analysis, anticoagulation was a protective factor for all-cause death. All of this evidence shows that antithrombotic therapy is relatively safe in patients with CMBs.

Cardio embolism prevention in patients with CMBs presents both risks and benefits. Anticoagulant therapy can reduce the risk of cardio embolism in patients with NVAF, but a risk of anticoagulant-related CH events remains, especially for AF patients with CMBs. Additionally, the bleeding risk should be considered, and appropriate antithrombotic strategies should be selected. Existing studies cannot provide clear guidance for primary cardio embolism prevention in patients with CMBs and AF. Traditional oral anticoagulants may increase the quantity of CMBs, especially in patients with cerebral CMBs and multiple CMBs. The current results for NOAC treatment in patients with CMBs are relatively optimistic. No difference was found in the incidence of adverse events between patients with CMBs who received antithrombotic therapy and those without CMBs in our study. However, the safety of antithrombotic therapy in patients with CMBs and NVAF still needs to be confirmed by large randomized controlled trials. In clinical practice, antithrombotic strategies used by physicians for primary prevention of cardio embolism in patients with CMBs tend to be conservative, whereas secondary prevention strategies for cardiac embolism in patients with CMBs tend to be more aggressive. This study found that antithrombotic therapy for secondary prevention of cardio embolism is relatively safe, which may provide guidance for primary prevention of cardiac embolism, thus reflecting the clinical significance of this study.

### Limitations

This study was conducted at a single center; thus, the generalizability of the results is limited. Importantly, however, our study was truly retrospective and included a real-world population with cardiogenic cerebral embolism with CMBs and NVAF. In the multivariate analysis, a parsimonious model was generated because of the small sample size, which may impact the strength of the associations between predictors and main adverse events. Large-scale, multicenter, and prospective studies should be conducted in the future, and subgroup analyses stratified by CMB variables should be performed.

## Conclusions

Antithrombotic therapy in patients with CMBs and cardiogenic cerebral embolism due to NVAF did not increase cerebral hemorrhage events and all-cause death. CMBs were probably not a crucial predictor of whether patients were prescribed antithrombotic medication. For patients with CMBs, antithrombotic therapy should be used with caution rather than contraindicated. Meanwhile, patients with CMBs and a hypertension history or cerebral hemorrhage history should receive a close follow-up because of the increasing risk of a cerebral hemorrhage event after antithrombotic therapy. Large-scale, multicenter, and prospective studies should be conducted in the future to investigate the significance of CMBs.

## Abbreviations

AF: Atrial fibrillation
CH: Cerebral hemorrhage
CI: Cerebral infarction
CMBs: Cerebral microbleeds
MRI: Magnetic resonance imaging
NIHSS: National Institutes of Health stroke scale
NOACs: Non-vitamin K antagonist oral anticoagulants
NVAF: Nonvalvular atrial fibrillation
SWI: Susceptibility-weighted imaging
TOAST: Trial of org 10,172 in acute stroke treatment
